# Histo-blood group antigens and rotavirus vaccine shedding in Nicaraguan infants

**DOI:** 10.1038/s41598-019-47166-9

**Published:** 2019-07-24

**Authors:** Filemón Bucardo, Yaoska Reyes, Ylva Rönnelid, Fredman González, Sumit Sharma, Lennart Svensson, Johan Nordgren

**Affiliations:** 1 Department of Microbiology, Faculty of Medical Science, National Autonomous University of Nicaragua, León (UNAN-León), León, Nicaragua; 2 Division of Molecular Virology, Department of Clinical and Experimental Medicine, Linköping University, Linköping, Sweden; 3 Department of Medicine, Karolinska Institute, Stockholm, Sweden

**Keywords:** Gastroenteritis, Live attenuated vaccines, Genetic markers

## Abstract

ABO, Lewis and secretor histo-blood group antigens (HBGA) are susceptibility factors for rotavirus in a P-genotype dependent manner and can influence IgA seroconversion rates following rotavirus vaccination. To investigate the association between HBGA phenotypes and rotavirus vaccine shedding fecal samples (n = 304) from a total of 141 infants vaccinated with Rotarix (n = 71) and RotaTeq (n = 70) were prospectively sampled in three time frames (≤3, 4–7 and ≥8 days) after first vaccination dose. Rotavirus was detected with qPCR and genotypes determined by G/P multiplex PCR and/or sequencing. HBGAs were determined by hemagglutination and saliva based ELISA. Low shedding rates were observed, with slightly more children vaccinated with RotaTeq (19%) than Rotarix (11%) shedding rotavirus at ≥4 days post vaccination (DPV). At ≥4 DPV no infant of Lewis A (n = 6) or nonsecretor (n = 9) phenotype in the Rotarix cohort shed rotavirus; the same observation was made for Lewis A infants (n = 7) in the RotaTeq cohort. Putative *in-vivo* gene reassortment among RotaTeq strains occurred, yielding mainly G1P[8] strains. The bovine derived P[5] genotype included in RotaTeq was able to replicate and be shed at long time frames (>13 DPV). The results of this study are consistent with that HBGA phenotype influences vaccine strain shedding as similarly observed for natural infections. Due to the low overall shedding rates observed, additional studies are however warranted.

## Introduction

Rotaviruses typically infect the gastrointestinal tract and are shed in very large amounts (>10^8^ genomes/gram of feces) for several days in feces of symptomatic and asymptomatic infants following natural infections^1–3^.

Rotavirus shedding is affected by host factors such as age and immunity, along with viral genetic and virulence factors. In a safety and immunogenicity trial of live, attenuated human rotavirus vaccine 89–12 (precursor to the Rotarix vaccine, genotype G1P[8]), rotavirus shedding after one dose was nil in adults, 15% in rotavirus IgA seropositive infants (2 to 12 year) and 70% in rotavirus IgA seronegative infants (6 to 26 weeks). Another interesting finding of two clinical trials with the Rotarix and 89–12 vaccines in Finland and USA, respectively, was the association between shedding and IgA seroconversion after vaccination in seronegative infants. 80% and 60% of the seronegative infants that shed rotavirus seroconverted after vaccination, respectively^4,5^. Although affected by detection methods and sampling times, studies using the Rotarix vaccine have found shedding rates generally ranging between 30–70% after one dose in infants^6^, with studies from Malawi, Mexico and Taiwan using PCR methods for detection having reported shedding rates between 30–90% ≥4 days post vaccination^7–9^.

The comprehension of how multivalent rotavirus vaccine prevent disease is more complex in terms of host susceptibility, viral replication and development of immune response. These vaccines contain multiple reassortant viruses derived from human and animal strains which may have limited permissiveness in the human host. Nearly one-third (7/22) of infants in USA (5 months–6 years) challenged with the Wistar Calf-3 strain (WC3), backbone of the RotaTeq vaccine, shed the virus^10^, However a similar study with the same strain performed in French infants, using a similar challenge dose (3 × l0^7^ pfu/mL), reported only 4.5% (1/22) of shedding while 88% developed neutralizing antibody response to the challenge serotype^11^. Pre-licensure studies reported higher viral shedding and transmission after vaccination with the old tetravalent rhesus rotavirus vaccine than with the current human pentavalent bovine-human reassortant vaccine (RotaTeq)^6^. A study from Taiwan using PCR methods for detection reported shedding rates for RotaTeq of 85–90%, 4–7 days post vaccination^7^.

Besides virulence factors and host immune response, genetic susceptibility to particular rotavirus genotypes may also affect virus shedding. Observational studies in humans indicate that Lewis B (LeB) secretor infants are significantly more susceptible to P[8] infections as compared to with non-secretors and/or infants with the Lewis A (LeA) phenotype^12–16^. Other studies have also suggested a role of ABO blood groups in susceptibility^17,18^. The cohorts in this study were previously investigated regarding seroconversion and showed that infants with the LeA phenotype did not exhibit IgA seroconversion (4-fold increase) after the first vaccine-dose of either Rotarix or RotaTeq vaccines^19^. Similarly, a Rotarix clinical trial in Pakistani infants reported limited IgA seroconversion (3-fold increase) in non-secretors after 2 vaccine doses^20^, and a recent study from Ghana reported similar results^21^. Moreover, a study from Malawi reported significantly less Rotarix vaccine shedding after first dose in non-secretors^9^, although no statistical difference (p > 0.05) regarding seroconversion was observed.

The role of HBGAs in RotaTeq vaccination has not been much investigated^19^, and might be complex since the vaccine contain the human P[8] genotype and 4 human-bovine reassortant strains of P[5] genotype which could imply a broader host range^22^. Thus, it is also of interest to investigate whether replication/shedding of the different strains and P-genotypes in the RotaTeq vaccine are associated with HBGA phenotypes.

Efficacy of the live rotavirus vaccines varies widely between populations, high income settings in Europe and North America having higher efficacy compared to Nicaragua^23^ and particularly sub-Saharan Africa^24^. As both vaccine efficacy and HBGA distribution differs markedly between different populations, a hypothesis raised is that less population susceptibility to the live vaccines will lead to less vaccine take and less subsequent protection; which could be one factor explaining the differences of vaccine efficacy.

Vaccine strain replication in the host most likely results in a more robust immune response, and hence vaccine shedding as a marker of vaccine take and efficacy needs to be further investigated. Our hypothesis is that the HBGA profile in infants influence vaccine replication as measured by presence of vaccine strains in stool after vaccination.

## Results

### No rotavirus shedding was observed in infants with the LeA phenotype at ≥4 DPV

At any sampled time frame, overall 16 (23%) of the 71 infants vaccinated with Rotarix and 33 (47%) of the 70 vaccinated with RotaTeq had at least one rotavirus-positive stool after vaccination. At ≥4 days post vaccination (DPV), also more children vaccinated with RotaTeq (19%) than with Rotarix (11%) shed rotavirus (Table 1). The distribution of Lewis phenotypes was similar in Rotarix (LeB = 73%, LeA-B- = 17% and LeA = 10%) and RotaTeq (LeB = 75%, LeA-B- = 15% and LeA = 10%) cohorts. At ≥4 DPV no rotavirus shedding was observed in LeA infants in either of the vaccine cohorts (Table 1). The distribution of secretor phenotype was also similar (86% and 85%) in both Rotarix and RotaTeq cohorts, respectively. All non-secretors from both cohorts (n = 19) were homozygous carriers of the FUT2 G428A (rs601338) nonsense mutation. In the Rotarix cohort, no rotavirus shedding was observed in non-secretors as compared to 13% in secretors. Similarly, fewer non-secretor infants vaccinated with RotaTeq shed rotavirus (10%, *RR* = 0.5 [95% CI: (0.07–3.3)]) as compared with secretors (21%) (Table 1). Blood group distribution was similar in Rotarix (O = 75%, A = 17%, B = 6%, AB = 2%) and RotaTeq (O = 66%, A = 21%, B = 13%, AB = 0) cohorts; this blood group distribution is typical for the region^25^. In the Rotarix cohort, shedding rates was highest for infants of blood group A (18%, RR = 1.7 [95% CI: 0.4–7.7]), whereas in the RotaTeq cohort, rotavirus shedding was lower (7%, *RR* = 0.3, [95% CI: 0.04–2.3) for infants of blood group A as compared to blood group O (30%) and B (22%) (Table 1). The distribution of Lewis and secretor phenotypes in children that abandoned the study in Rotarix and RotaTeq cohorts was not significantly different.

**Table 1:** Association between HBGA phenotypes and rotavirus positivity in infants at ≥4 days post vaccination (DPV) with RotaTeq or Rotarix.

HBGAs	Infants RV-positive at ≥4 DPV					
	Rotarix			RotaTeq		
	All infants	Rotavirus, n (%)	RR (95% CI)	All infants	Rotavirus, n (%)	RR (95% CI)
**Lewis phenotype**						
LeB	46	6 (13)	1.0	51	10 (20)	1.0
LeA-B-	11	1 (9)	0.7 (0.09–5.2)	10	3 (30)	1.5 (0.5–4.5)
LeA	6	0 (0)	0.0 (NA)	7	0 (0)	0.0 (NA)
**Secretor Phenotype**						
Secretor	54	7 (13)	1.0	58	12 (21)	1.0
Non-secretor	9	0 (0)	0.0 (NA)	10	1 (10)	0.5 (0.07–3.3)
**Blood groups**						
O	47	5 (11)	1.0	45	10 (22)	1.0
A	11	2 (18)	1.7 (0.4–7.7)	14	1 (7)	0.3 (0.04–2.3)
B	4	0 (0)	0.0 (NA)	9	2 (22)	1.0 (0.3–3.8)
AB	1	0 (0)	NA	0	0 (0)	NA
**All HBGAs**	63^a^	7^b^ (11)		68^c^	13 (19)	

^a^In the Rotarix cohort 8 children did not provide samples at ≥4DPV, all secretors, of which 7 were LeB and 1 was Le A-B-.

^b^Vaccine shedding in these children was confirmed by the Rotarix specific NSP2 assay, sequencing (n = 3) and G and P genotyping by gel electrophoresis (n = 4).

^c^In the RotaTeq cohort 2 children did not provide samples at ≥4DPV, both were secretors, LeB.

### Rotavirus genotyping

A total of 19 (13%) of the 146 samples collected from infants vaccinated with Rotarix were rotavirus-positive as compared with 46 (29%) of the 158 samples collected from RotaTeq vaccinated infants. Genotyping and sequencing analysis of 5 rotavirus-positive samples from the Rotarix cohort confirmed the presence of the G1P[8] Rotarix strains (Table 2). For the RotaTeq cohort, a total of 12 of the 17 rotavirus-positive samples collected at ≥4 DPV could be genotyped. Of these, 3 were RotaTeq derived G1P[8], 4 were RotaTeq derived P[5] with unknown G-type, 2 were RotaTeq derived G6P[8], 2 were RotaTeq derived G4P[5], thus all of them confirmed as RotaTeq strains (Table 2). Of note, 1 genotype G4P[8] was observed in a non-secretor infant of LeA-B- phenotype but limited PCR product (Ct = 35) precluded sequence analysis and could not be confirmed as RotaTeq derived.

**Table 2:** Rotavirus genotypes observed in stools at ≥ 4 DPV in Nicaraguan infants of different HBGA phenotypes following vaccination with RotaTeq or Rotarix.

Vaccine	Code	HBGA phenotype	4 to 7 DPV^a^	Ct2^b^	RV genotype	≥8 DPV^a^	Ct3	RV Genotype	Nucleotide homology
RV5	024	Secretor:LeB:O	6	31	**G1P[8]** ^**c**^	8	27	**G1P[8]**	>98% with RotaTeq VP7, VP4, VP6:I2 and VP3:M2.
	033	Secretor:LeB:A	7	23	G6P[8]	8	22	**G1P[8]**	>98% with RotaTeq VP7, VP4, VP6:I2 and VP3:M2.
	047	Non-secretor:LeA-B-:O	6	35	**G4P[8]**	9	40	Negative	Not sequenced.
	049	Secretor:LeB:B	4	33	GntP[5]^d^	12	40	Negative	Not sequenced
	048	Secretor:LeB:O	10	31	nt	13	34	GntP[5]	Not sequenced
	050	Secretor:LeB:B	4	30	G6P[8]	10	32	Nt	>98% with RotaTeq VP4. VP7 not sequenced
	054	Secretor:LeB:O	3	32	GntP[5]	8	40	Negative	Not sequenced
	068	Secretor:LeB:O	3	37	Negative	20	30	G4P[5]	>98% with RotaTeq VP7 and VP6:I2. VP4 not sequenced.
	073	Secretor:LeA-B-:O	NSA			16	28	G4P[5]	>98% with RotaTeq VP7 and VP4.
	082	Secretor:LeB:O	4	33	GntP[5]	NSA^e^			>98% with RotaTeq VP6:I2.
RV1	103	Secretor:LeB:O	5	24	G12P[8]	NSA			G12 Nicaraguan strain
	105	Secretor:LeB:O	6	40	Negative	9	32	G12P[8]	G12 Nicaraguan strain
	109	Secretor:LeB:A	6	34	G12P[8]	8	40	Negative	G12 Nicaraguan strain
	113	Secretor:LeB:O	4	37	Negative	9	27	G12P[8]	G12 Nicaraguan strain
	131	Secretor:LeB:O	6	27	G1P[8]	NSA			G1 Rotarix
	134	Secretor:LeB:A	4	27	G1P[8]	17	32	G1P[8]	G1 Rotarix
	149	Secretor:LeB:O	5	31	G12P[8]	13	34	G12P[8]	G12 Nicaraguan strain
	170^f^	Secretor:LeB:O	8	40	G1P[8]	11	40		Not sequenced
	174	Secretor:LeB:O	6	29	G1P[8]	12	29	G1P[8]	G1 Rotarix

^a^Days post-vaccination, intended 2nd and 3rd sampling time frame, respectively.

^b^Cycle threshold.

^c^Bold letter indicate possible reassortant strain.

^d^“nt” stands for not typed after examination for the most common G- (1, 2, 3, 4, 9 and 12) and P-types (P[8], P[4], P[6] and P[5]) in a nested multiplex PCR.

^e^“NSA” stands for “no sample available” because mothers did not collect sample at that point.

^f^This child had a conflicting sample NSP3-negative (Ct 40), NSP2-positive (Ct 36).

### Putative *in vivo* gene reassortment among RotaTeq strains

Interestingly, putative gene reassortment among RotaTeq strains occurred commonly in the subset examined, yielding the G1P[8] genotype in two infants (Table 2). Sequences analysis of the VP6 gene (codes 024 and 033) from the putative *in-vivo* reassortment show high nucleotide homology (99%) with the bovine VP6 (GU565043) present in the WI79-4 RotaTeq strain. Similarly, VP3 sequences of the 024 putative *in-vivo* reassortant strain (genotype G1P[8]) showed 99% of nucleotide homology with VP3 of bovine origin present in RotaTeq (BrB-9 [G4P[5]], WI78-8 [G3P[5]], and WI79-4 [G6P[8]]) (Table 2) and was not similar to the VP3 gene of RotaTeq strain W179-9 (G1P[5]), showing that the VP7 gene of G1P[5] RotaTeq strain likely reassorted to the WI79-4 backbone. The viral load of the G1P[8] reassortants was high (Ct, 27 and 22), extended until at least 8 DPV and occurred among infants of secretor, LeB and blood groups O and A phenotypes. To support the possibility of gene reassortment *in vivo*, virus in stool specimen collected from a child 8 days post RotaTeq vaccination (sample ID 33) was propagated for 2 passages in MA-104 cells and subsequently subjected to PCR genotyping. The results show detection of G1 and G6 and P[8] genotypes. Genotype P[5] was not detected thus indicating reassortment of the G6P[8] and G1P[5] strains in the vaccine.

### Concomitant natural rotavirus infections during vaccination with Rotarix

Further examination of the samples collected at ≥4 DPV in the Rotarix cohort using a real-time PCR assay specific for Rotarix NSP2 showed discordant result in samples for 6 children (NSP2-negative, NSP3-positive and vice-versa) suggesting shedding of non-Rotarix strains or sensitivity differences between the NSP3 and NSP2 assays. VP7 and VP4 genotyping by sequencing or semi-nested multiplex PCR of the samples with discordant results demonstrated rotavirus G12P[8] (n = 5, NSP2 negative) and G1P[8] (n = 1, NSP2 positive) genotype. G12P[8] had high nucleotide homology with wild type rotavirus circulating in Nicaragua at the same time frame (Table 2). The 5 children with confirmed G12P[8] infections were excluded for HBGAs analysis. G12P[8] infections were only observed among infants of secretor and LeB phenotypes.

## Discussion

In this study, we have investigated if HBGAs affect viral replication of Rotarix and RotaTeq vaccine strains in infants by determining rotavirus shedding at different time points after first dose of vaccination. The rationale for testing vaccine shedding after first dose is that natural infections and seroconversion to rotavirus occur early in life in Nicaragua^26^. This increases the risk for natural rotavirus infection during the course of vaccination, which might complicate the analysis of the effect of HBGAs on vaccine shedding. Also, the association between HBGA and vaccine shedding might be affected if children have already been immunized with one dose of vaccine. A study in Malawi observed lower rates of Rotarix shedding in non-secretors after first dose, but no differences in shedding rates between secretor phenotypes was observed after the second dose^9^.

No infants of LeA and non-secretor phenotypes in the Rotarix cohort shed vaccine strains at ≥4 DPV; and no child with LeA phenotype shed in the RotaTeq cohort. A major limitation of our study is that the number of LeA and non-secretors were low; and given the unexpectedly low rates of shedding in general, this reduced statistical power. No HBGA phenotype was as such significantly associated with reduced shedding (p > 0.05) which warrants a careful interpretation of the results. The observations are however consistent with previous studies that have shown less susceptibility to P[8] rotavirus infection in individuals of the LeA and non-secretor phenotype^13–16,27^. *In vitro* studies have also demonstrated that P[8] rotavirus does not bind to LeA but to secretor antigens, such as H type 1 and LeB^12,28,29^.

Previous *in vitro* studies have found that P[8] binding to types A/AB and O saliva was significantly higher than to type B saliva^12,17^. Furthermore, a recent study from Egypt found that rotavirus gastroenteritis was significantly more prevalent among infants with blood group A as compared with group B, but this study did not include rotavirus genotyping^18^. However, several other studies have failed to find any association between ABO and rotavirus gastroenteritis^30,31^, or susceptibility to different genotypes^9^. In this study, no rotavirus shedding was observed in infants of blood group B vaccinated with Rotarix but the number of blood group B children in the Rotarix cohort was too low to draw any conclusions. However, the result is consistent with seroconversion data of the studied cohorts, where after vaccination with Rotarix, blood group B children seroconverted to a significantly lesser extent (5%) compared to secretor-positive children with blood groups A (26%) and O (27%)^19^. A previous study from Pakistan found lower seroconversion rates in non-O blood groups^20^. However, other studies have not found any association between seroconversion and/or vaccine shedding of Rotarix in relation to ABO^9,21^.

In the RotaTeq cohort higher rates of rotavirus shedding at ≥4 DPV was found in infants of blood group B (22%) and O (22%), compared to blood group A (7%).

More studies are needed regarding ABO as putative susceptibility factors for rotavirus and whether these can account for differences between shedding pattern between the two vaccines.

Individual differences regarding stimulation of immune response, virus shedding and protection to rotavirus disease have been observed in several studies, but rarely considered in context of host genetic factors, such as HBGAs. Kapikian and coworkers described in the early 80’s that only 67% of adult volunteers inoculated with the Wa strain (G1P[8]) developed serologic evidence of infection and 23% did not shed rotavirus nor developed a significant increase in neutralizing antibodies titers against the inoculating strain^32^. Bernstein and coworkers reported 70% shedding after first inoculation of the vaccine candidate 89-12, precursor of the Rotarix vaccine, in seronegative North American infants^5^. A more recent study in non-Hispanic North American infants showed that 79% of the Rotarix vaccinated infants shed the vaccine strain at >3 DPV^33^. These studies demonstrate a lack of rotavirus shedding in approximately 20–30% of seronegative infants vaccinated with Rotarix, suggesting that this subgroup was not susceptible to vaccination with P[8] rotavirus. Interestingly, approximately 25% of infants in North America are non-secretors, suggesting that the secretor phenotype may account for this pattern of non-susceptibility to the vaccines, as observed for natural infections of P[8] strains^14,16^. In this study, non-secretors infants vaccinated with Rotarix did not shed rotavirus at ≥4 DPV whereas less shedding (10%, n = 1) was observed among non-secretors from the RotaTeq cohort.

Rotavirus shedding was observed at any time frame in 23% and 47% of the infant vaccinated with Rotarix and RotaTeq, respectively, and only in 11% and 19% at ≥4 DPV. These are low vaccine shedding rates compared to previous studies in Taiwanese and North American infants^7,33^. A previous study from Malawi, also found relatively low shedding rates for Rotarix ≥4 DPV after first dose (30%). A possible explanation for this difference might be the high rates of rotavirus IgA pre-vaccination reported for the studied cohorts (56% and 40%, respectively)^19^, a common observation in countries from Latin America, Africa and Asia^19,26,34^. Bernstein and coworkers observed limited shedding in rotavirus-seropositive (15%) infants as compared with rotavirus seronegative (70%)^5^ which has also been reported elsewhere^4^. The fact that rotavirus vaccine shedding is limited after second dose in Rotarix and RotaTeq vaccinated infants is indicative that early acquired immunity effectively affects shedding^7,35^. Accordingly, analysis of serum rotavirus IgA pre-vaccination in a subset of children in this study with available serological data (n = 41 and 53 for Rotarix and RotaTeq cohorts, respectively), demonstrated less rotavirus vaccine shedding ≥4 DPV in seropositive children (0%, 0/14), as compared to seronegative children (22%, 6/27) in the Rotarix cohort. No such effect was observed for the RotaTeq cohort, with 33% (4/12) shedding in seropositive children compared to 20% (8/41) in seronegative children. The proportion of non-secretors was similar in seropositive and seronegative children in both cohorts (14% and 15%; 8% and 12%, for Rotarix and RotaTeq respectively).

A further explanation for higher shedding in the RotaTeq cohort could be that the pre-existing IgA response was less effective against RotaTeq, which is antigenically more diverse that Rotarix, as also supported by the fact that the proportion of shedding between IgA seropositive and seronegative infants pre-vaccination was similar in the RotaTeq cohort. The selection of method to screen for rotavirus vaccine shedding may contribute to differences in shedding rates between studies. Concomitant natural rotavirus infections may also be a confounder for studies using methods not specific for vaccine strains. This would be particularly important in countries with high rotavirus transmission or studies carried out during rotavirus epidemics.

Molecular characterization of rotavirus positive stool showed several interesting findings. Six of the characterized rotavirus strains from 5 children in the Rotarix cohort were G12P[8] wild type viruses, a genotype that was causing a large rotavirus epidemic in Nicaragua during the time frame of collection for this cohort^36^. The vaccine strain G1P[8] was not observed with the methods used in G12 positive stool, possibly due to higher viral load of G12P[8] viruses. This suggest that studies investigating vaccine take or response after full course of vaccination may yield a biased result due to early infections. Natural rotavirus infections during vaccination has also been reported in RotaTeq vaccinated populations^37^.

The segmented genome of rotavirus enables gene reassortment. Following vaccination with RotaTeq a reassortment event may occur if 2 vaccine strains infect a single cell. *In vivo*, reassortment among RotaTeq strains seems to be possible and give rise mainly to G1P[8] viruses^38–40^. In the current study, putative G1P[8] reassortant strains were observed in 2 infants at >5 DPV. It is likely that these strains has an evolutionary advantage, since it contains the human P[8] genotype of VP4 found to recognize the LeB, type A and H type1 antigens in humans^12,41^. These G1P[8] reassortant can occasionally cause gastroenteritis symptoms in vaccine recipients who may transmit the virus to close contacts, as described previously^39,42,43^. Of further note, P[5] RotaTeq strains were detected as late as 13–20 DPV in LeB, LeA-B-, secretors and blood group A and B infants, thus demonstrating prolonged replication and shedding of the bovine P[5] rotavirus. There are no documented cases of natural infection in human with wild type P[5] rotavirus, as far as we have found, and it is thus interesting to speculate that bovine VP4 of P[5] genotype included in the RotaTeq vaccine may recognize these human HBGAs or other glycans. Related to this was the findings that the G6P[5] parental bovine strain in RotaTeq is sialic acid independent^22^, a common characteristic of human rotavirus^44^. The shedding of P[5] rotavirus derived from RotaTeq in children with gastroenteritis have been reported previously^39,45^.

The results of this study are consistent with that HBGA phenotype influences vaccine strain shedding as similarly observed for natural infections of the P[8] genotype. However, given the unexpected low overall shedding rates observed, additional studies are warranted. Differences in susceptibility pattern between the two vaccines also warrants further investigation, especially with regards to the bovine P[5] component in the RotaTeq vaccine.

## Materials and Methods

### Study site and subjects

This study was carried out in 3 health territories from Leon (Perla Maria, Ruben Dario and Antenor Sandino), Nicaragua. The expectancy of live birth children in these territories was 772 per year and the rotavirus vaccine coverage was approximately 95%. The study sample was randomly selected from the newborn registers (N = 228) by estimating 70% of the primary outcome (rotavirus vaccine shedding), with 95% confidence interval (CI). For several reasons, including migration, missing address, and rejection to participate only 141 Nicaraguan infants (aged 2 months) participated in the study. Children were enrolled at the vaccination units before receiving the first dose of either RotaTeq (September 2013 to July 2014) or Rotarix vaccines (March to July 2015), as part of a project investigating factors influencing rotavirus vaccine take in Nicaraguan infants^19^. In this study, we intended to collect 3 stool samples from each child, the first sample at ≤3 days post vaccination (DPV) to stablish the shedding baseline, second sample at 4 to 7 DPV to match with the replication peak and ≥8DPV to investigate long term shedding. The 71 infants vaccinated with Rotarix provided 146 stools samples, distributed as follows: 47 at ≤3 DPV, 50 between 4 to 7 DPV (median = 6, IQR: 5–7) and 49 at ≥8DPV (median = 12, IQR: 10–14). Likewise, the 70 infants vaccinated with RotaTeq provided 158 samples, distributed as follows: 56 at ≤3 DPV, 38 between 4 to 7 DPV (median = 5, IQR: 4–7) and 64 at ≥8DPV (median = 11, IQR: 9–14). No child provided multiple samples for a given time frame and 16 children from the Rotarix cohort provided only 1 sample, mainly at ≤3DPV.

### Sampling

Stool samples were collected from diaper in sterile plastic containers by the infants´ mothers at the indicated time frames and transported at 4 °C to the at UNAN-León. After enrolment, blood and saliva samples were collected by a nurse for examination of HBGA. Upon arrival to the laboratory, a 10% weight/volume stool suspension was prepared and stored in a freezer (−20 °C) for viral RNA purification. ABO blood phenotyping was performed on the day of collection, while saliva was stored at −20 °C for HBGAs subsequent examination. None of the collected samples was diarrheic.

### Ethics

The protocol and questionnaire used in this study were reviewed and approved by the Ethical committee for Biomedical Research of UNAN-León (Acta No. 18, 2012) and the methods performed in accordance with guidelines and regulations. Written informed consent was obtained from the parents of all children included in this study.

### RNA purification and cDNA synthesis

Viral RNA was extracted from 200 µl of 10% stool suspensions using High Pure Viral RNA Kit (Roche Applied Science, Indianapolis, USA) following the manufacturer’s instructions. A total of 50 µl of RNA was collected and stored at −20 °C for rotavirus detection by real time PCR. Reverse transcription (RT) was carried out as described previously^46^.

### Real time PCR assays for rotavirus detection

The primers and probes based on the NSP3 gene described by Freeman and coworkers were used for rotavirus screening^47^. In brief, 2 µl of cDNA was added to a reaction mixture consisting of 10 µl of iTaq Universal Probe mix (BIO-RAD Laboratories, Hercules, CA), 0.8 µl (10 pmol) of each NVP3-FDeg and NVP3-Rprimers, and 0.3 µl (10 pmol) of NVP3-Probe, 6.1 µl of RNAse free water, to final volume of 20 µl. The real-time PCR reactions were performed in a 96-well reaction plate using the BIO-RAD CFX96 Manager 2.1 System (BIO-RAD Laboratories, Hercules, CA). PCR was performed under the following conditions: 95 °C for 3 min, followed by 40 cycles of 95 °C for 5 s, 60 °C for 30 s. A value of cycle threshold (Ct) <36 was considered rotavirus-positive. Samples collected from infants vaccinated with Rotarix were also examined with a qPCR assay specific for the Rotarix strain^48^. No sensitivity and specificity data for the NSP3 assay was described, but detection limit was stated as 44 copies of NSP3 per reaction. For the NSP2 assay the sensitivity and specificity reported was 100% and 99%, respectively with a detection limit at 2 NSP2 copies per reaction^47,48^.

### Rotavirus G and P genotyping

For all rotavirus-positive samples collected ≥4 DPV, a hemi-nested multiplex PCR was applied for G and P typing. Rotavirus G (G1, G2, G3, G4, G9 and G12) and P (P[4], P[6] and P[8]) genotypes were determined following a nested PCR method as described elsewhere^49,50^.

### RotaTeq P[5] detection

For all rotavirus-positive samples in the RotaTeq cohort collected ≥4 DPV, a P[5] specific PCR was applied as the commonly used P-typing primers do not include P[5]. Primers F1P5 and R1P5 were designed to target a consensus region in the VP4 gene of several sequences derived from bovine strains WC3 present in the RotaTeq vaccine and reported in the GenBank (accession numbers GU565055, GU565066, GU565077 and GU565088). The F1P5 (5′-ATATCAGCGGCGGTGCTAAA-3′) and R1P5 (5′-CGTTTCCACCTTGTACGCGA-3′) primers were used to develop a new PCR method by using the RotaTeq vaccine as reference. In brief, 2.5 µl of cDNA was added to a reaction mixture consisting of Ready-To-Go PCR bead (GE Healthcare, Uppsala, Sweden), 0.75 µl (10 pmol) of each F1P5 and R1P5 and 21 µl of RNAse free water, to final volume of 25 µl. PCR was performed under the following conditions: 95 °C for 5 min, followed by 40 cycles of 95 °C for 30 s, 50 °C for 30 s, 72 °C for 30 s and final extension of 72 °C for 7 min. PCR products were analyzed by gel electrophoresis using 2% agarose gel and ethidium bromide staining, an amplicon of 185-bp was indicative of P[5].

### Sequencing of VP7, VP6, VP4 and VP3 genes

In order to confirm shedding of vaccine derived strains, all rotavirus-positive samples collected at ≥4 DPV were further analyzed with Sanger sequencing when there was sufficient rotavirus cDNA (usually (Ct ≤35). The VP7 (881-bp), VP6 (379-bp) and VP4 (876-bp) gene segments of rotavirus were amplified by PCR using methods and primers described elsewhere^49,51^. To study reassortant virus origin in RotaTeq-positive samples (≥4 DPV), a VP3 gene segment (229 bp) was amplified by using primers manually designed in this study VP3F2: 5′-CGCATGTAGATCGGCAAAGG-3′ and VP3R2: 5′-ATCCGGCACCATGGAATCTG-3′. The partial VP7, VP6, VP4, and VP3 gene amplicons were subsequently sequenced by Sanger sequencing (Macrogen, Amsterdam).

### Isolation of RotaTeq virus from stool

To support the possibility of gene reassortment *in vivo*, virus in stool was cultivated in MA-104 cells and subsequently subjected to G and P genotyping as previously described with minor modifications^52^.

### ABO, Lewis and secretor phenotyping and genotyping

ABO blood typing, Lewis and secretor phenotyping on saliva and genotyping on the *FUT2* G428A (rs601338) nonsense mutation was performed previously as described^19^.

### Statistical analysis

Statistical analyses were performed in SPSS 14.0 and OpenEpi (Version 3.01) available online. The number of rotavirus-positive infants at ≥4 DPV was stratified according to Lewis, secretor and blood group HBGAs. The category from each HBGA with the highest number of rotavirus-positive at ≥4DPV was used as reference for relative risk (*RR*) calculation with 95% confidence intervals (CI). Rotavirus positive samples with confirmed wild type infection were excluded for analysis regarding rotavirus positivity at ≥4 DPV.
